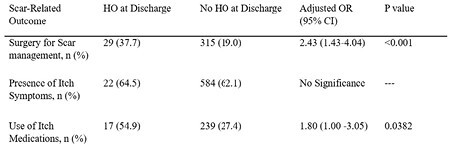# 709 Scar-Related Outcomes in Burn Patients with Heterotopic Ossification

**DOI:** 10.1093/jbcr/irae036.254

**Published:** 2024-04-17

**Authors:** Danielle J Sim, Keith T Kuo, Julie A Caffrey

**Affiliations:** Johns Hopkins University School of Medicine, Baltimore, MD; Johns Hopkins Department of Plastic and Reconstructive Surgery, Baltimore, MD; Johns Hopkins University School of Medicine, Baltimore, MD; Johns Hopkins Department of Plastic and Reconstructive Surgery, Baltimore, MD; Johns Hopkins University School of Medicine, Baltimore, MD; Johns Hopkins Department of Plastic and Reconstructive Surgery, Baltimore, MD

## Abstract

**Introduction:**

Heterotopic ossification (HO) and aberrant scar formation are pathologic wound healing processes that may occur after burns. Hypertrophic scars occur in up to 70% of burn patients and can negatively affect quality of life. HO is characterized by the development of osseous lesions in the soft tissue and occurs in about 3.5% of burn patients, causing significant pain and delays in rehabilitation. Burns can incite a prolonged systemic inflammatory response involving innate and adaptive immune mediators, and this process has been shown to play a role in the development of both hypertrophic and keloid scars as well as HO. TGF-β1-producing macrophages are also implicated in excess extracellular matrix deposition and osteogenic differentiation in pathologic scarring and HO, respectively. Given the similar biologic mechanisms involved in these sequelae, this study aims to identify the clinical impact of HO on scarring in burn patients.

**Methods:**

The Burn Model System (BMS) National Database was queried from August 1, 2015 - May 2, 2022 to identify adult patients (=>18 years) with and without HO at discharge. Patients with a burn etiology other than thermal, acid, or electrical were excluded. RStudio 4.3.0 (Posit, Boston, MA) was used for statistical analyses.

**Results:**

In this sample, 135 (3.3%) patients had HO at discharge. The HO group was younger and exhibited a greater proportion of flame burns compared to non-HO group. The HO sample also had a larger average %TBSA and a higher incidence of other injuries.

On multivariate logistic regression, after controlling for sex, age, other injuries, and etiology, HO patients were more likely to have surgery for scar management at least once during the 24-month follow-up period (OR 2.43, 95% CI: 1.43—4.04, p< 0.001). HO patients were also more likely to report use of medications for itch symptoms (OR 1.80, 95% CI: 1.00—3.05, p = 0.0382). There was no difference in the incidence of pruritis between the two groups.

**Conclusions:**

Patients with HO sustained more severe injuries than non-HO patients and required more frequent surgical and medical scar management. The increased use of itch medications suggests that HO patients experienced more severe symptoms despite having a similar incidence in pruritis overall. Therefore, HO is associated with worse scar outcomes after burns, potentially through immune-related mechanisms involved in both processes. Pruritis may also be related to a prolonged systemic inflammatory response as it is stimulated by cytokine IL-31 via induction of TGF-β1. Future research should identify methods of attenuating the immune response to prevent these sequelae without compromising wound healing or infection control.

**Applicability of Research to Practice:**

This study advocates for increased awareness of symptomatic scarring in patients with HO. It also indicates the need to develop an immunomodulatory approach for reducing the risk of pathologic wound healing sequelae.